# An Ionic Liquid Electrolyte Additive for High-Performance Lithium–Sulfur Batteries

**DOI:** 10.3390/ma16237504

**Published:** 2023-12-04

**Authors:** Zeliang Guan, Ling Bai, Binyang Du

**Affiliations:** MOE Key Laboratory of Macromolecular Synthesis and Functionalization, Department of Polymer Science & Engineering, Zhejiang University, Hangzhou 310027, China; 21929001@zju.edu.cn (Z.G.); 12129056@zju.edu.cn (L.B.)

**Keywords:** lithium–sulfur battery, ionic liquid, electrolyte, additive

## Abstract

With the development of mobile electronic devices, there are more and more requirements for high-energy storage equipment. Traditional lithium-ion batteries, like lithium–iron phosphate batteries, are limited by their theoretical specific capacities and might not meet the requirements for high energy density in the future. Lithium–sulfur batteries (LSBs) might be ideal next-generation energy storage devices because they have nearly 10 times the theoretical specific capacities of lithium-ion batteries. However, the severe capacity decay of LSBs limits their application, especially at high currents. In this study, an ionic liquid (IL) electrolyte additive, TDA^+^TFSI, was reported. When 5% of the TDA^+^TFSI additive was added to a traditional ether-based organic electrolyte, the cycling performance of the LSBs was significantly improved compared with that of the LSBs with the pure traditional organic electrolyte. At a rate of 0.5 C, the discharge specific capacity in the first cycle of the LSBs with the 5% TDA^+^TFSI electrolyte additive was 1167 mAh g^−1^; the residual specific capacities after 100 cycles and 300 cycles were 579 mAh g^−1^ and 523 mAh g^−1^, respectively; and the average capacity decay rate per cycle was only 0.18% in 300 cycles. Moreover, the electrolyte with the TDA^+^TFSI additive had more obvious advantages than the pure organic ether-based electrolyte at high charge and discharge currents of 1.0 C. The residual discharge specific capacities were 428 mAh g^−1^ after 100 cycles and 399 mAh g^−1^ after 250 cycles, which were 13% higher than those of the LSBs without the TDA^+^TFSI additive. At the same time, the Coulombic efficiencies of the LSBs using the TDA^+^TFSI electrolyte additive were more stable than those of the LSBs using the traditional organic ether-based electrolyte. The results showed that the LSBs with the TDA^+^TFSI electrolyte additive formed a denser and more uniform solid electrolyte interface (SEI) film during cycling, which improved the stability of the electrochemical reaction.

## 1. Introduction

With the development of mobile electronic equipment and clean energy vehicles, traditional lithium-ion batteries can no longer meet people’s demand for future energy storage equipment because of their low theoretical specific capacity and energy density. Lithium–sulfur batteries (LSBs) have become next-generation energy storage equipment because of their extremely high theoretical specific capacity (1675 mAh g^−1^) and energy density (2600 wh kg^−1^) [1,2]. At present, the electrolyte in LSBs generally uses organic ethers, such as 1,3-dioxolane (DOL), 1,2-dimethoxyethane (DME), or tetraethylene glycol dimethyl ether (TEGDME) [3,4]. Organic ether-based electrolytes have the advantages of low prices, high ionic conductivities, and low viscosities, but they have many problems, such as a severe polysulfide shuttle effect [5,6], which will lead to the actual discharge specific capacity of the LSBs being lower than the theoretical discharge specific capacity and the charge–discharge not being stable enough during long-term cycling. Ionic liquids (ILs) have many advantages, like low polysulfide solubility [7,8], high ionic conductivity [9,10], and non-flammability [11,12,13]. Therefore, many kinds of ILs have been reported as electrolytes [14,15,16]. For example, Jun-Woo Park et al. reported a room-temperature IL N,N-diethyl-N-methyl-N-(2-methoxyethyl) ammonium bis(trifluoromethanesulfonyl) amide ([DEME] [TFSI]) [17]. When the [DEME] [TFSI] IL was used to replace the ether-based electrolyte TEGDME, the battery had a higher discharge specific capacity of 600 mAh g^−1^ after 100 cycles at 1/12 C and a more stable Coulombic efficiency, nearly 100%. This can be attributed to the weak Lewis alkalinity of TFSI^−^ anions and the low donor capacity of the room-temperature IL, resulting in the poor solubility of polysulfides. Yang et al. reported an IL N-methoxyethyl-N-methylpyrrolidinium bis(trifluoromethanesulfonyl)imide (Pyr_1,2O1_TFSI)-based electrolyte and used it in LSBs [18]. Pyr_1,2O1_TFSI and DOL were mixed at a ratio of 65:35 (*w*/*w*) to obtain a mixed electrolyte. The LSBs assembled with the mixed electrolyte had a good capacity performance of 650 mAh g^−1^ after 100 cycles at 0.2 C, and the Coulombic efficiency was higher than 99%, which was owing to the restrained polysulfide dissolution in the electrolyte. However, there are few reports on the use of ILs as electrolyte additives. Additives account for a small part of the electrolyte, but they can have a great impact on the performance of the battery [19,20]. Lithium nitrate is a common additive [21,22], but it is hard to maintain the stability of the surface film during cycling owing to the lack of toughness of the film containing inorganic substances [23]. So many salts and organic compounds have been used as additives in electrolytes [24,25,26,27,28,29]. For instance, Vu et al. reported aluminum phosphate (AlPO_4_) as an additive in a mixed liquid electrolyte; the specific capacity of LSBs containing 1.0 wt.% AlPO_4_ remained at 453 mAh g^−1^ after 100 cycles at 1.0 C [30]. The authors proved that the improved performance could be attributed to the adsorption of lithium polysulfide on the surface of the AlPO_4_. Lu et al. reported lithium bis(fluorosulfonyl)imide (LiFSI) as a functional additive in the fluorinated electrolyte for rechargeable LSBs. The electrolyte with 1.0 wt.% of the LiFSI additive delivered a high initial specific capacity of 1254 mAh g^−1^ and a capacity retention of up to 78% after 50 cycles at 0.2 C [31]. The additive component improved the ion transport and slightly restrained the polysulfide dissolution of the electrolyte. The anions of lithium salts can form a stable SEI film, but few studies have been conducted on the capture of additives forming SEI films.

**Scheme 1:**
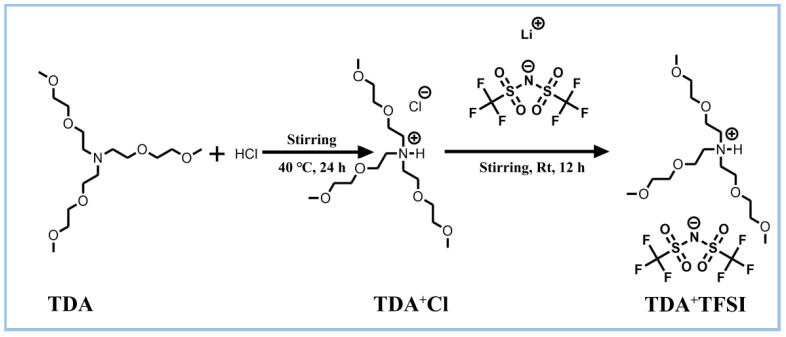
Synthesis routine of TDA^+^TFSI.

In this work, we report a kind of IL named TDA^+^TFSI as an electrolyte additive, which was synthesized by the quaternization reaction of tris(dioxa-3,6-heptyl) anime (TDA) and hydrochloric acid (HCl) followed by anion exchange with lithium bis(trifluoromethanesulfonyl)imide (LiTFSI), as shown in Scheme 1. The electrochemical performances of the LSBs using electrolytes with different TDA^+^TFSI additive contents were tested. When 5% of the TDA^+^TFSI additive was added to the ether-based organic electrolyte, the cycling performance of the LSBs was significantly improved. The LSBs with 5% of the TDA^+^TFSI electrolyte additive exhibited high specific capacities of 1167 and 924 mAh g^−1^ at 0.5 and 1.0 C, respectively, and good capacity retentions of 579 and 428 mAh g^−1^ after 100 cycles and 523 and 399 mAh g^−1^ after 300 cycles, respectively, at 25 °C. At the same time, the Coulombic efficiency of the LSBs with 5% of the TDA^+^TFSI electrolyte additive was more stable than that of the LSBs with the traditional organic ether-based electrolyte. The results of the SEM and electrochemical tests suggest that the LSBs with 5% of the TDA^+^TFSI electrolyte additive formed a denser and more uniform SEI film during cycling, which improved the stability of the electrochemical reaction.

## 2. Materials and Methods

### 2.1. Materials

Tris(dioxa-3,6-heptyl) anime (TDA, 95%) was purchased from HEOWNS Biochem Technologies LLC, Tianjin, China. Super P, sublimed sulfur, and hydrochloric acid (HCl, 36.0–38.0%) were purchased from Sinopharm Chemical Reagent Co., Ltd., Shanghai, China. Lithium bis(trifluoromethanesulfonyl)imide (LiTFSI, 99.9%) was purchased from Shanghai Macklin Biochemical Co., Ltd., Shanghai, China. Polyvinylidene difluoride (PVDF), N-methylpyrrolidone (NMP), 1,3-dioxolane (DOL, 99.5%), 1,2-dimethoxyethane (DME, 99.5%), and lithium nitrate (LiNO_3_, 99%) were purchased from Aladdin Chemical Ltd., Shanghai, China. DOL and DEM were soaked with 4A molecular sieves to remove water before use.

### 2.2. Fabrication of IL Additive TDA^+^TFSI

TDA (17.97 g, 0.056 mol) and HCl (30.6 mL, 2 M) were mixed in a 100 mL round bottom flask and stirred at 40 °C for 24 h. Then, the solvent and redundant HCl were removed by rotary evaporation. Liquid products (19.75 g, 0.055 mol) were obtained and named as TDA^+^Cl. After that, 17.33 g (0.060 mol) of LiTFSI and 10 mL of deionized water were added to the TDA^+^Cl, and the mixture was stirred for 12 h and then layered. The lower liquid layer was collected as the final product, TDA^+^TFSI. The presence of Cl^−^ ions in the TDA^+^TFSI was checked by adding a AgNO_3_ aqueous solution to a small portion of the TDA^+^TFSI. No precipitate of AgCl was observed, indicating that the TDA^+^TFSI did not contain any Cl^−^. The 4A molecular sieves were used to remove any trace of water so that the TDA^+^TFSI could be used as an electrolyte additive in LSBs. The TDA^+^TFSI was stored in a glovebox before use. To confirm that there was no trace of water in the TDA^+^TFSI, after the 4A molecular sieve was added, a piece of Li metal was directly placed in the TDA^+^TFSI to make sure that there was no reaction between the Li metal and possible trace of water.

### 2.3. Preparation of C/S Complex and Cathode

The C/S complex was prepared through the melt-infiltration method [32,33,34]. Specifically, 210 mg of sublimed sulfur powder and 90 mg of Super P were mixed and ground in a mortar. Then, the mixture was put into a pipe furnace and heated to 150 °C at a heating rate of 5 °C min^−1^. After being held at 150 °C for 6 h to ensure the sulfur powder had infiltrated the pores of the Super P, the obtained C/S complex was slowly cooled to room temperature. The cathode was made as follows: the C/S complex, Super P, and PVDF, in a weight ratio of 7:2:1, were dissolved in NMP to form a uniform slurry and then the slurry was coated on aluminum foil. The cathode was dried under vacuum at 70 °C for 12 h and then transferred to a glovebox before use. The diameter and area of the aluminum foil were 12 mm and 1.13 cm^2^, respectively, and the sulfur content of the cathode was 0.90 ± 0.10 mg cm^−2^.

### 2.4. Physical Characterization

^1^H-NMR spectra were recorded on a Bruker 400 MHz spectrometer (Bruker Corp., Karlsruhe, Germany) with chloroform-d (CDCl_3_) containing 3% tetramethylsilane (TMS) as the solvent. The sample concentration was approximately 15 mg mL^−1^. Fourier-transform infrared (FTIR) spectra were recorded using a Bruker TENSOR II spectrometer (Bruker Corp., Karlsruhe, Germany). The mass spectrum of the TDA^+^TFSI was recorded on an Agilent quadrupole time-of-flight LC/MS G6545 mass spectrometer (Agilent, Santa Clara, CA, USA). The surface morphology of the lithium metal was observed using a Hitachi S-4800 scanning electron microscope (SEM, Hitachi, Ltd., Tokyo, Japan). The batteries were disassembled in a glovebox, and the lithium metal was cleaned with DOL/DME (volumetric ratio: 1/1) to remove the remaining electrolyte.

### 2.5. Electrochemical Characterization

CR2032-type coin cells were assembled in an Ar-filled glovebox for electrochemical characterizations and charge–discharge cycle tests. The separator used glass microfiber filters purchased from Whatman. The surface morphology and thickness of the glass fiber separator were characterized using SEM (Figure S1a,b, Supporting Information). The electrolyte/sulfur (E/S) ratio was 100 µL mg^−1^. The charge–discharge cycling performance of the LSBs with different TDA^+^TFSI additive contents was characterized using the LAND battery testing system (CT2001A, Wuhan LAND Electronic Co., Ltd., Wuhan, China) at 25 °C, and the voltage range was 1.8–3.0 V. The electrochemical stability window of the TDA^+^TFSI was measured using an electrochemical workstation (CHI660E, CH Instruments Inc., Shanghai, China). Linear sweep voltammetry measurements were performed from the open-circuit voltage to 5.0 V and 1.0 V vs. Li/Li^+^ at a potential scan rate of 1 mV s^−1^. The conductivities (σ) of the electrolytes with different TDA^+^TFSI additive contents were measured using the same instrument and a CR2032-type coin cell, of which both the anode and cathode were stainless-steel pistons. The impedances of the lithium symmetrical coil cells and full coil cells were measured using the same instrument in the frequency range from 100 mHz to 100 kHz at a perturbation amplitude of 5 mV.

### 2.6. Preparation of Electrolytes with Different TDA^+^TFSI Additive Contents

The electrolytes containing ionic liquids were prepared by adding TDA^+^TFSI to a mixed solution of DOL and DME. The formulae for the electrolytes with different TDA^+^TFSI additive contents are shown in Table 1.

## 3. Results and Discussion

**Figure 1:**
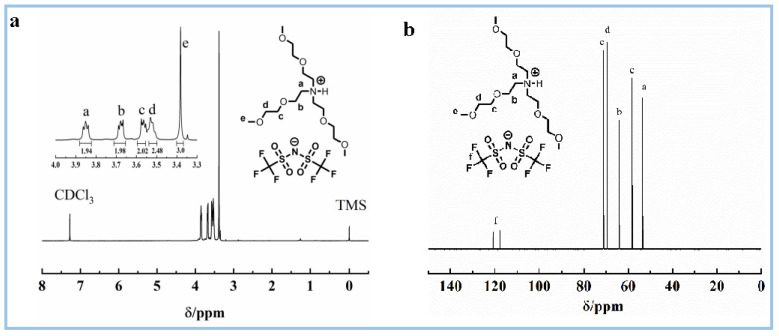
(**a**) ^1^H-NMR spectrum of TDA^+^TFSI in chloroform-d; (**b**) ^13^C-NMR spectrum of TDA^+^TFSI in chloroform-d.

The TDA^+^TFSI additive was synthesized by the quaternization reaction of TDA and HCl, followed by anion exchange with LiTFSI. Figure 1a shows the ^1^H-NMR spectrum of TDA^+^TFSI, which confirms the chemical structure of TDA^+^TFSI. The chemical shifts of the peaks are assigned as follows: δ 3.94 (q, *J* = 5.3 Hz, 6H) (N^+^-CH_2_-CH_2_), 3.64–3.58 (m, 6H) (CH_2_-CH_2_-OCH_3_), 3.54 (t, *J* = 4.8 Hz, 6H) (N^+^-CH_2_-CH_2_), 3.51–3.46 (m, 6H) (CH_2_-CH_2_-OCH_3_), and 3.31 (s, 9H) (CH_2_-CH_2_-OCH_3_). The ^13^C-NMR spectral analysis of TDA^+^TFSI is also provided in Figure 1b, which confirmed the synthesis of the TDA^+^TFSI molecule. The chemical shifts of the peaks are assigned as follows: ^13^C-NMR (101 MHz, D_2_O) δ 120.90, 117.73, 71.05, 69.59, 64.03, 58.09, and 53.55.

**Figure 2:**
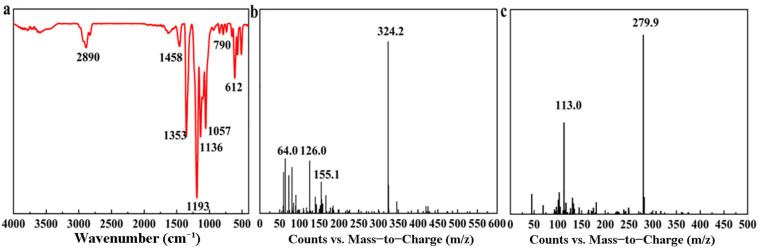
(**a**) The FT−IR spectrum of TDA^+^TFSI; (**b**) the mass spectrum of TDA^+^TFSI in the positive mode; (**c**) the mass spectrum of TDA^+^TFSI in the negative mode.

FT−IR spectroscopy was used to characterize the structure of TDA^+^TFSI (Figure 2a). The result showed that the characteristic absorption peak at 2890 cm^−1^ is attributed to the C-H stretching vibration, and the peak at 1458 cm^−1^ corresponds to the C-H in-plane bending vibration. There are characteristic absorption peaks at 1353 cm^−1^ and 1136 cm^−1^, which belong to the S=O symmetrical telescopic vibration and asymmetrical telescopic vibration of TDA^+^TFSI. Meanwhile, the characteristic absorption peaks at 1193 cm^−1^, 1057 cm^−1^, 790 cm^−1^, and 612 cm^−1^ correspond to the C-O-C vibration, S-N-S stretching vibration, CF_3_ vibration, and O=S=O vibration, respectively [35]. Figure 2b,c show the mass spectra of TDA^+^TFSI. The positive and negative ions of TDA^+^TFSI show obvious peaks in the mass spectra. The strong ion peaks in the positive mode could be identified as N^+^H(CH_2_CH_2_OCH_2_CH_2_OCH_3_)_3_ (at *m*/*z* = 324.3), and other peaks correspond to fragments. The strong ion peaks in the negative mode could be identified as N^−^(SO_2_CF_3_)_2_ (*m*/*z* = 279.9), and the peak at *m*/*z* = 113.0 represents an interior label.

**Figure 3:**
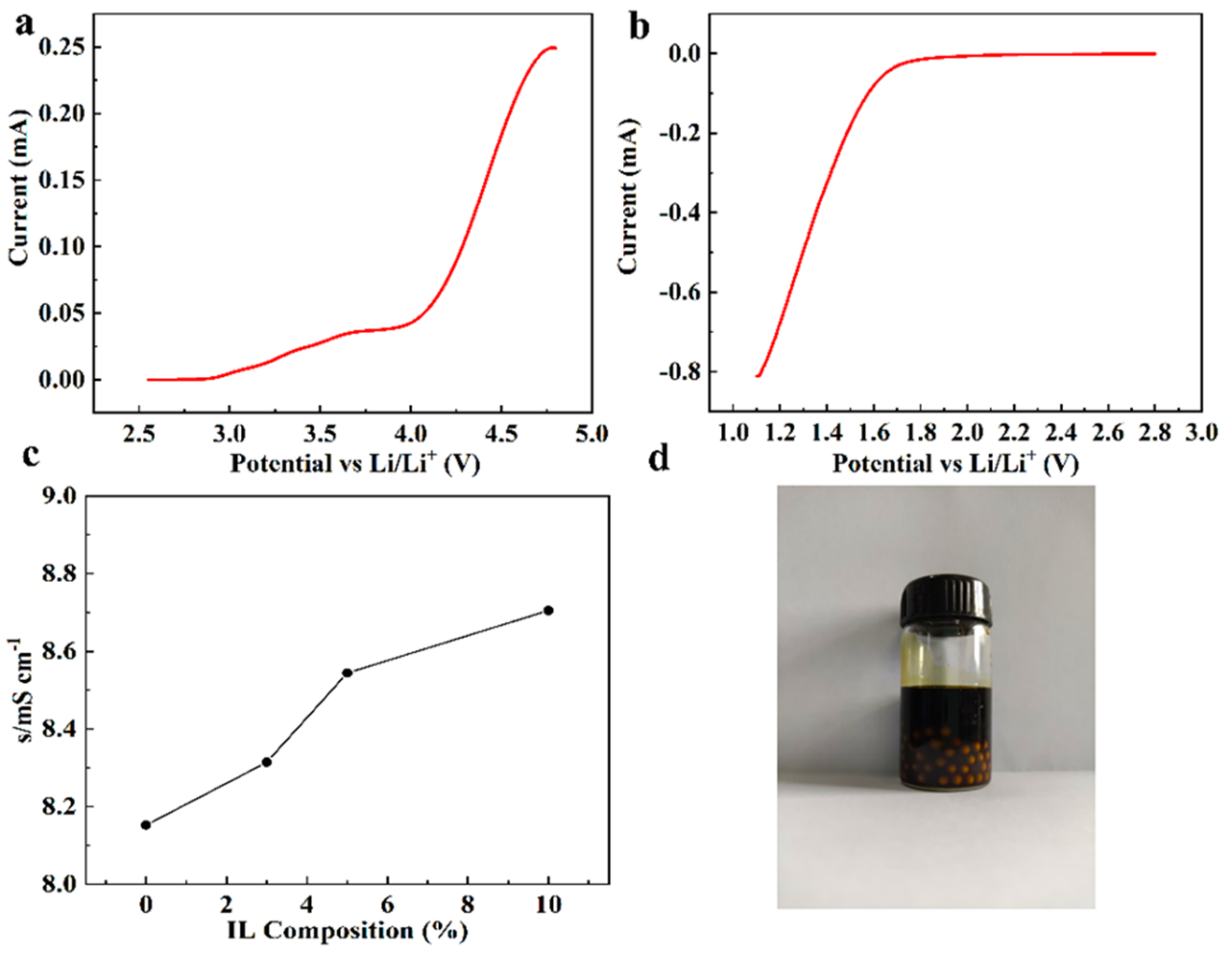
(**a**) LSV curve of TDA^+^TFSI from 2.5 V to 5.0 V. (**b**) LSV curve of TDA^+^TFSI from 2.8 V to 1.0 V. (**c**) Ionic conductivities of electrolytes with different TDA^+^TFSI additive contents (the test temperature is 25 °C). (**d**) Photo of TDA^+^TFSI.

As shown in Figure 3a,b, TDA^+^TFSI was stable in the electrochemical window of 1.6 V–4.0 V at 25 °C, which is wider than the cutoff voltage of LSBs, i.e., from 1.8 V to 3.0 V. The cathodic current below 1.6 V vs. Li^+^/Li could be attributed to the reductive decomposition of the quaternary ammonium salt structure of TDA^+^TFSI, indicating that the additive forms an SEI film. The ionic conductivities of the electrolytes with different TDA^+^TFSI additive contents were characterized, and the results are shown in Figure 3c. The ionic conductivity increased with increasing content of the TDA^+^TFSI additive. With increasing TDA^+^TFSI additive content, neutral organic molecules tend to substitute the TFSI^−^ because of the stronger interaction between Li^+^ and organic molecules [36]. Therefore, the proportions of Li^+^-DME and Li^+^-DOL increased, while the coordination between lithium and TFSI^−^ anions decreased. This yields a higher fraction of “free” TFSI^−^ ions, leading to higher ionic conductivity [37]. The high ionic conductivity is beneficial to the electrochemical reaction of the LSBs. Figure 3d is the photograph of TDA^+^TFSI, showing a dark brown liquid characteristic.

**Figure 4:**
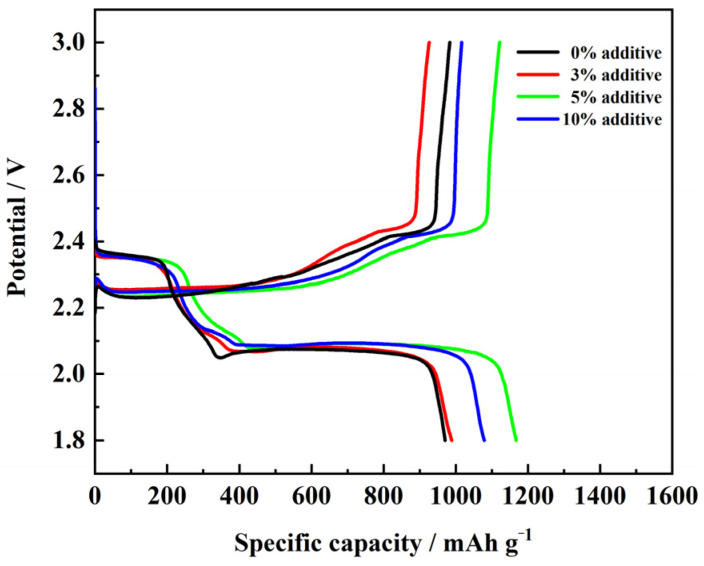
Charge–discharge curves in the first cycle of LSBs using electrolytes with different TDA^+^TFSI additive contents; the charge–discharge current density was 0.5 C.

To evaluate the performances of different TDA^+^TFSI additive contents in LSBs, coin cells were assembled using carbon Super P/S composites with an ~71 wt.% sulfur content as the cathode (Figure S2). The galvanostat charge-discharge of LSBs with different TDA^+^TFSI additive contents were tested at 25 °C with a current density of 0.5 C. Figure 4 shows the charge–discharge curves in the first cycle of the LSBs assembled with 0%, 3%, 5%, and 10% TDA^+^TFSI electrolyte additives. The curves of all the LSBs using the electrolytes with the TDA^+^TFSI additive had two discharge plateaus. The first discharge plateau is located at 2.3 V–2.4 V, which corresponds to the reduction of the active substance, S_8_, to high-order polysulfides (S_8_^2−^ and S_6_^2−^). The second discharge plateau is located at 2.0 V–2.1 V, which corresponds to the higher-order polysulfides (S_8_^2−^ and S_6_^2−^) further obtaining electrons to become lower-order polysulfides (S_4_^2−^ and S_2_^2−^) [38,39]. Similarly, there are two charge plateaus in the curves of these systems. The first charge plateau is located at 2.2 V–2.3 V. This plateau represents the reverse process of the corresponding reaction for the second discharge plateau, which corresponds to the oxidation of the final discharge product, Li_2_S, to form low-order polysulfides (S_4_^2−^ and S_2_^2−^). The second charge plateau is located at 2.3 V–2.4 V, this plateau represents the reverse process of the corresponding reaction for the first discharge plateau, which corresponds to low-order polysulfides further losing electrons to form high-order polysulfides (S_8_^2−^ and S_6_^2−^). These results indicate that the electrolytes with the TDA^+^TFSI additive had no effect on the electrochemical reaction of the LSBs. 

**Figure 5:**
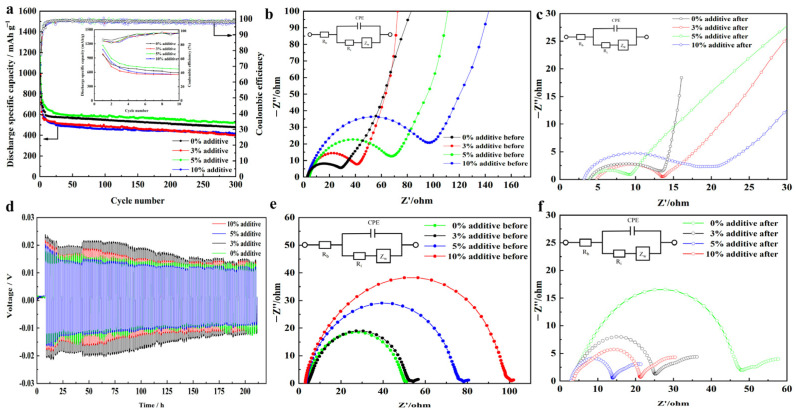
(**a**) Long-term cycling performances of LSBs using electrolytes with different TDA^+^TFSI additive contents at a charge–discharge current density of 0.5 C. (Inset: amplified cycling performance curves). (**b**) EIS of LSBs using electrolytes with different TDA^+^TFSI additive contents before 300 cycles. The inset shows the analog circuit. (**c**) EIS of LSBs using electrolytes with different TDA^+^TFSI additive contents after 300 cycles. The inset shows the analog circuit. (**d**) The voltage profiles of the Li||Li symmetrical cells using electrolytes with different TDA^+^TFSI additive contents at 0.1 mA cm^−2^ and a stripping/plating capacity of 1 mAh cm^−2^. (**e**) EIS of lithium symmetrical cells using electrolytes with different TDA^+^TFSI additive contents before the cycling test. The inset shows the analog circuit. (**f**) EIS of lithium symmetrical cells using electrolytes with different TDA^+^TFSI additive contents after the cycling test. The inset shows the analog circuit.

Figure 5a shows the long-term cycling performance of the LSBs using electrolytes with different TDA^+^TFSI additive contents at 25 °C and a current density of 0.5 C. The specific capacities of the LSBs with TDA^+^TFSI additive contents of 0%, 3%, 5%, and 10% were 971, 988, 1167, and 1079 mAh g^−1^ in the first cycle, respectively. After 100 cycles, the corresponding specific capacities decayed to 550, 461, 579, and 483 mAh g^−1^, respectively. After 300 cycles, the corresponding specific capacities decayed to 478, 416, 523, and 400 mAh g^−1^, respectively. Among the different LSBs, the LSBs using the electrolyte with 5% of the TDA^+^TFSI additive showed the excellent electrochemical performance during the whole cycle. The average capacity decay rate per cycle for the electrolyte with 5% of the TDA^+^TFSI additive was only 0.18% in 300 cycles, and the Coulombic efficiency of the LSBs using the electrolyte with 5% of the TDA^+^TFSI additive was 97–99%. However, the specific capacities of the electrolytes with 3% and 10% TDA^+^TFSI additive contents were no better than that of the electrolyte without the TDA^+^TFSI additive. This may be due to the different effects of the electrolytes with different TDA^+^TFSI additive contents on the formation of SEI films.

To verify the effects of different TDA^+^TFSI additive contents on SEI films, the impedances of LSBs using electrolytes with different TDA^+^TFSI additive contents were tested before and after 300 cycles at 0.5 C, and the results are shown in Figure 5b,c, respectively. The analog circuit shown in the inset of Figure 5b was used to simulate the experiment to obtain the impedance spectrum. The bulk resistance (*R*_b_) (i.e., the intercept between the semicircle and real axis) values of the LSBs using electrolytes with 0%, 3%, 5%, and 10% of the TDA^+^TFSI additive were 3.1, 3.9, 4.4, and 3.5 Ω, respectively [40,41], while the interfacial resistance (*R*_i_) (i.e., the diameter of the semicircle) values of the LSBs were 25.5, 39.3, 67.4, and 81.4 Ω, respectively. After 300 cycles were run at 0.5 C, the corresponding *R*_b_ values of the LSBs using electrolytes with 0%, 3%, 5%, and 10% of the TDA^+^TFSI additive became 4.2, 5.1, 3.6, and 3.1 Ω, respectively, and the *R*_i_ values of the LSBs became 7.6, 7.6, 5.4, and 15.9 Ω, respectively. The *R*_b_ values of the cells with 0% of the additive and 3% of the additive increased, while the *R*_b_ values of the cells with 5% of the additive and 10% of the additive decreased. This may be due to the reduction in the internal resistance of the LSBs during cycling with increasing TDA^+^TFSI additive content. Therefore, the battery can provide more stored electric energy to the external circuit. In addition, the change in *R*_b_ before and after cycling can be almost ignored, while the *R*_i_ value is substantially reduced. This can be attributed to the fact that the TDA^+^TFSI additives in the electrolyte participate in the reaction to form SEI films during cycling, which accelerates the charge transfer. The LSBs using the electrolyte with 5% of the TDA^+^TFSI additive had the lowest *R*_i_ value after cycling, which is consistent with the best performance of the 5% TDA^+^TFSI additive in the cycle test. These results might suggest that the SEI film formed with the 5% TDA^+^TFSI additive was denser and more uniform than the other SEI films.

The cycling performances of lithium symmetrical cells using electrolytes with different TDA^+^TFSI additive contents and the impedances before and after 100 cycles were also studied. Figure 5d shows the cycling performances of lithium symmetrical cells at 0.1 mA cm^−2^ and a Li capacity of 1 mAh cm^−2^. The electrolytes with 0%, 3%, 5%, and 10% of the TDA_+_TFSI additive had initial overpotentials of 19, 24, 20, and 22 mV, respectively. In addition, the overpotentials of the electrolytes with 0%, 3%, and 5% of the TDA^+^TFSI additive showed significant fluctuations after 50 h, which can be attributed to the destruction of SEI films and unstable interfacial channels between the metallic lithium and electrolyte. On the contrary, the electrolyte with 5% of the TDA^+^TFSI additive still maintains a relatively smooth voltage plateau and a low overpotential over 200 h, indicating that the electrolyte with 5% of the TDA^+^TFSI additive is conducive to stabilize Li anodes and lower the occurrence of polarization during continuous Li stripping/plating. These results suggest that the electrolyte with 5% of the additive forms the most stable and uniform SEI film on the Li metal anode. Figure 5e,f show the impedance spectra of the lithium symmetrical cells before and after 100 cycles. The *R*_b_ values of the lithium symmetrical cells using electrolytes with 0%, 3%, 5%, and 10% of the TDA^+^TFSI additive were 3.9, 4.7, 3.5, and 3.2 Ω before cycling, respectively, and the *R*_i_ values were 45.4, 48.2, 72.5, and 95.2 Ω, respectively. After 100 cycles, the *R*_b_ values became 3.6, 3.5, 3.1, and 3.5 Ω, respectively, and the *R*_i_ values became 43.5, 21.6, 11.0, and 18.5 Ω, respectively. In addition, the relationship between the impedance and overpotential of the symmetrical batteries with or without the TDA^+^TFSI additive is as follows: On the one hand, owing to the higher viscosity of the electrolyte containing 5% of the TDA^+^TFSI additive compared with the electrolyte containing 0% of the TDA^+^TFSI additive, the permeability between the electrolyte with 5% of the additive and the electrode is relatively incomplete at the beginning of the cycle, resulting in a higher impedance value of the battery. This leads to the battery with 5% of the TDA^+^TFSI additive having a high initial overvoltage. The complete contact between the electrolyte and lithium metal electrode during continuous Li stripping/plating results in a decrease in the impedance value and a decrease in the overpotential. On the other hand, the electrolyte containing 5% of the TDA^+^TFSI additive is conducive to the formation of stable SEI films and can improve electrode interfaces, thereby reducing the interfacial impedance and charge-transfer resistance. The electrolyte with 0% of the additive has a low viscosity that enables the electrolyte to fully contact the electrode, resulting in minimal changes in the impedance value of the battery before and after cycling. However, owing to the instability of the SEI film formed by the electrolyte containing 0% of the TDA^+^TFSI additive, the SEI film continuously dissolves and reforms during the Li stripping/plating process, which leads to the continuous consumption of lithium nitrate in the electrolyte and causes overpotential fluctuations [22]. This result is basically consistent with the impedance change before and after the cycling of the LSBs; that is, the *R*_b_ values of the lithium symmetrical cells change slightly before and after cycling, while the *R*_i_ values decrease significantly after cycling. This phenomenon is also due to the formation of an SEI film on the lithium sheet surface during cycling. It is worth noting that the *R*_i_ value of the electrolyte without the additive changed little before and after cycling, which indicates that the TDA^+^TFSI additive in the electrolyte played a key role in the formation of the SEI film.

**Figure 6:**
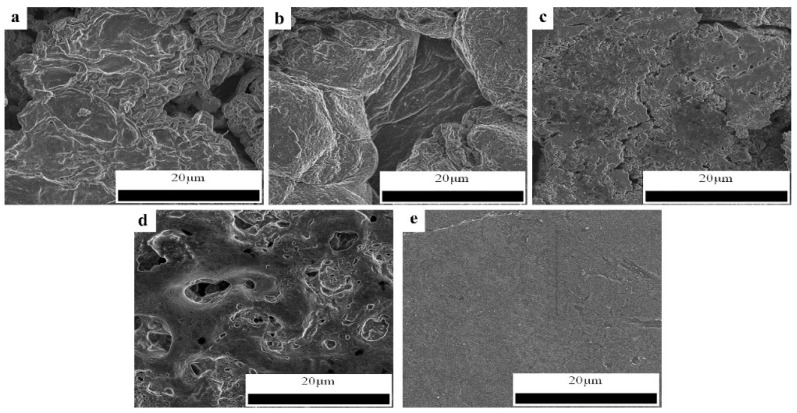
SEM images after 300 cycles at 0.5 C for lithium sheets of LSBs using electrolytes with different TDA^+^TFSI additive contents: (**a**) 0% additive, (**b**) 3% additive, (**c**) 5% additive, and (**d**) 10% additive and (**e**) fresh lithium sheet.

To further examine the difference between the SEI films formed by different electrolytes, the LSBs using electrolytes with different TDA^+^TFSI additive contents were disassembled after 300 cycles at 0.5 C, and the surface morphologies of the lithium sheets were observed using SEM. Figure 6a–d show the surface morphologies of the lithium sheets after cycling using electrolytes with 0%, 3%, 5%, and 10% of the TDA^+^TFSI additive, respectively. For comparison, the surface morphology of a fresh lithium sheet is shown in Figure 6e. The surface of the fresh lithium sheet is smooth and flat, while the surfaces of the lithium sheets after charging and discharging appear to have a layered structure. This layered structure is the SEI film formed on the surface of the lithium sheet during the electrochemical reaction. Compared with the SEI films on the surfaces of the lithium sheets using electrolytes with 3%, 5%, and 10% of the TDA^+^TFSI additive, the SEI film on the surface of the lithium sheet using the electrolyte with 0% of the TDA^+^TFSI additive has more layered structures, indicating that the TDA^+^TFSI additive contributes to the formation of a uniformly dense SEI film. Among the different electrolyte additive contents, the 5% TDA^+^TFSI additive content produced a lithium sheet that had the flattest and most uniform surface without any obvious hollowing, although obvious hollowing is observed on the surface of the lithium sheet using the electrolyte with a TDA^+^TFSI additive content of 3%. This may be because the content of the TDA^+^TFSI additive in the electrolyte is too low, resulting in an SEI film that is insufficient to evenly cover the entire lithium sheet’s surface. However, when the content of the TDA^+^TFSI electrolyte additive was increased to 10%, significant holes appeared on the surface of the lithium sheet, which is the result of the excessive growth of the SEI film caused by the excess additive in the electrolyte. The increase in the SEI film thickness forms an inert layer, leading to a reduced charge-transfer rate of the LSBs. This result is consistent with that of the cycling test: the smoother and denser the SEI film, the higher the specific capacity of the corresponding LSB.

**Figure 7:**
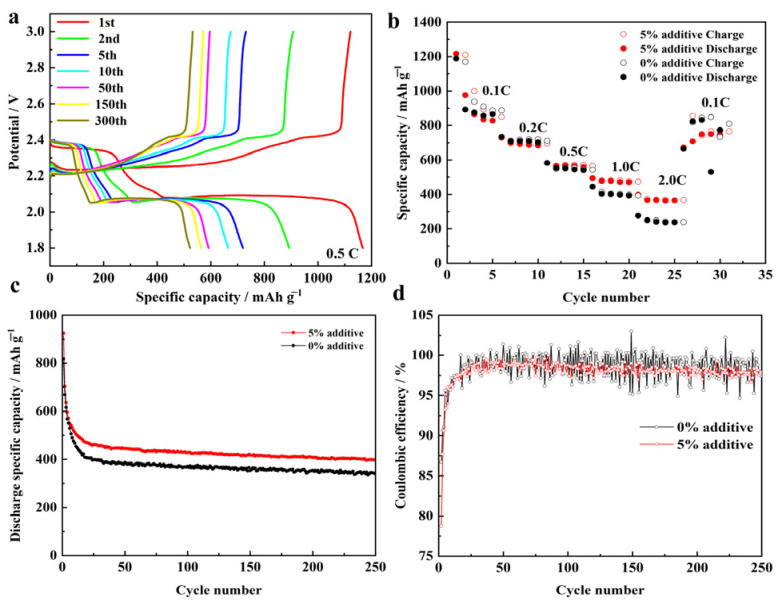
(**a**) Charge–discharge curves of the LSB using the electrolyte with 5% of the TDA^+^TFSI additive in different cycles at a current density of 0.5 C. (**b**) Rate performances of LSBs using electrolytes with 5% of the TDA^+^TFSI additive and without the additive. (**c**) Cycling performances of LSBs using electrolytes with 5% of the TDA^+^TFSI additive and without the additive at charge–discharge current densities of 1.0 C. (**d**) The corresponding Coulombic efficiencies of the LSBs using electrolytes with 5% of the TDA^+^TFSI additive and without the additive at a current density of 1.0 C.

The charge–discharge curves of the electrolytes with 5% and 0% of the TDA+TFSI additive in different charge–discharge cycles are shown in Figure 7a and Figure S3, respectively. The charge and discharge specific capacities of the two types of LSBs decreased with increasing number of cycles, but a clear charge–discharge plateau can still be observed, indicating that the addition of 5% TDA^+^TFSI to the electrolyte does not affect the electrochemical stability of the battery during the cycling process. The gradient rate capabilities of the LSBs using the electrolytes with 0% and 5% of the TDA^+^TFSI additive were investigated at 25 °C via the sequential variation in the current densities. As shown in Figure 7b, when the charge–discharge current was less than 0.5 C, there was little difference between the discharge specific capacities of the electrolytes with and without the additive. However, when the charge–discharge current was higher than 0.5 C, the electrolyte containing 5% of the TDA^+^TFSI additive obviously had a higher discharge specific capacity than the electrolyte without the additive. This result indicates that the 5% TDA^+^TFSI additive in the electrolyte forms a stable SEI film on the lithium sheet’s surface, which makes the electrochemical reaction more stable, and this effect becomes more obvious with increasing charge–discharge current. In addition, when the charge–discharge current is recovered to 0.1 C, the specific discharge capacity of the LSB is basically the same as that at the beginning, indicating that the LSB with 5% of the TDA^+^TFSI additive in the electrolyte has an excellent rate capability.

The cycling performances of the LSBs using electrolytes with or without the TDA^+^TFSI additive were tested at a high charge–discharge current density (1.0 C), and the results are shown in Figure 7c. The discharge specific capacity of the LSB using the electrolyte with 5% of the TDA^+^TFSI additive decreases from 924 mAh g^−1^ in the first cycle to 428 mAh g^−1^ in the 100th cycle and 399 mAh g^−1^ in the 250th cycle. At the same time, the Coulombic efficiency of the LSB using the electrolyte with 5% of the TDA^+^TFSI additive is always maintained at 97–100%. Although the discharge specific capacity of the LSB using the electrolyte without the additive decreases from 817 mAh g^−1^ in the first cycle to 372 mAh g^−1^ in the 100th cycle and 339 mAh g^−1^ in the 250th cycle, the Coulombic efficiency of the LSBs using the electrolyte without the additive is 95–102%. The specific capacity of the LSBs with the electrolyte containing 5% of the TDA^+^TFSI additive is always higher than that of the LSBs with the electrolyte without the additive, which is consistent with our observational results of the rate performance. In addition, it is worth noting that the Coulombic efficiency fluctuation of the LSBs with the electrolyte containing 5% of the TDA^+^TFSI additive is much smaller than that of the LSBs without the electrolyte additive (Figure 7d) because the TDA^+^TFSI additive in the electrolyte formed a more stable SEI film during the electrochemical reaction, which improved the stability of the lithium-ion plating and stripping. In addition, the large fluctuation in the Coulombic efficiency of the LSB using the electrolyte without the additive represented the severe polysulfide shuttle during cycling [42]. By contrast, the LSB with the TDA^+^TFSI additive formed an SEI film that could effectively prevent direct contact between the polysulfides and lithium sheet, thereby inhibiting the shuttle effect of the polysulfides. 

## 4. Conclusions

In summary, a new type of ionic liquid, TDA^+^TFSI, was synthesized and used as an electrolyte additive for LSBs. The cycling performance of the LSBs using the electrolyte with 5% of the TDA^+^TFSI additive was significantly improved. The discharge specific capacity of the first cycle was 1167 mAh g^−1^, and the residual specific capacities after 100 and 300 cycles were 579 mAh g^−1^ and 523 mAh g^−1^, respectively, at 0.5 C. The average capacity decay rate per cycle was only 0.18% in 300 cycles, which is significantly higher than that of the LSB using the electrolyte without the TDA^+^TFSI additive. Moreover, the LSBs using the electrolyte with 5% of the TDA^+^TFSI additive had more obvious advantages at a high charge–discharge current (1.0 C). On the one hand, the discharge specific capacity of the LSB with 5% of the TDA^+^TFSI electrolyte additive was higher than that of the LSBs without the additive. On the other hand, the LSB with 5% of the TDA^+^TFSI electrolyte additive had a more stable Coulombic efficiency and a higher cycling stability than the LSBs without the TDA^+^TFSI additive. This is because the TDA^+^TFSI additive helps to form more dense and more uniform SEI films during cycling, thereby improving the stability of the electrochemical reactions. This work provides guidance for developing highly stable ionic liquid electrolytes for high-performance LSBs.

## Figures and Tables

**Table 1:** Electrolyte formulations with different TDA^+^TFSI additive contents.

Samples	Volume Ratio of TDA^+^TFSI (vol%)	Volume Ratio of DOL (vol%)	Volume Ratio of DME (vol%)	LiTFSI Concentration (mol/L)	LiNO_3_ Concentration (mol/L)
0% IL	0	50	50	0.5	0.2
3% IL	3	48.5	48.5	0.5	0.2
5% IL	5	47.5	47.5	0.5	0.2
10% IL	10	45	45	0.5	0.2

## Data Availability

The data are contained within the article.

## Supplementary Materials

The following supporting information can be downloaded at: https://www.mdpi.com/article/10.3390/ma16237504/s1, Figure S1: (a) SEM images of the top surface for glass fiber separator, (b) Cross-section of the glass fiber separator; Figure S2: The TG curve of Super P/S; Figure S3: The charge-discharge curve of LBSs used electrolyte with 0% TDA^+^TFSI additive in different cycle, the current density is 0.5 C.

## References

[1] Bruce P.G., Freunberger S.A., Hardwick L.J., Tarascon J.M. (2012). Li-O_2_ and Li-S Batteries with High Energy Storage. Nat. Mater..

[2] Manthiram A., Fu Y.Z., Chung S.H., Zu C.X., Su Y.S. (2014). Rechargeable Lithium-Sulfur Batteries. Chem. Rev..

[3] Peled E., Sternberg Y., Gorenshtain A., Lavi Y. (1986). Lithium-Sulfur Battery-Evaluation of Cioxolane-Based Electrolytes. J. Electrochem. Soc..

[4] Zheng J., Fan X.L., Ji G.B., Wang H.Y., Hou S., DeMella K.C., Raghavan S.R., Wang J., Xu K., Wang C.S. (2018). Manipulating Electrolyte and Solid Electrolyte Interphase to Enable Safe and Efficient Li-S Batteries. Nano Energy.

[5] Conder J., Bouchet R., Trabesinger S., Marino C., Gubler L., Villevieille C. (2017). Direct Observation of Lithium Polysulfides in Lithium-Sulfur Batteries using Operando X-ray Diffraction. Nat. Energy.

[6] Mikhaylik Y.V., Akridge J.R. (2004). Polysulfide Shuttle Study in the Li/S Battery System. J. Electrochem. Soc..

[7] Wang L.N., Liu J.Y., Yuan S.Y., Wang Y.G., Xia Y.Y. (2016). To Mitigate Self-Discharge of Lithium-Sulfur Batteries by Optimizing Ionic Liquid Electrolytes. Energy Environ. Sci..

[8] Zhang S.S. (2013). New Insight into Liquid Electrolyte of Rechargeable Lithium/Sulfur Battery. Electrochim. Acta.

[9] Dokko K., Tachikawa N., Yamauchi K., Tsuchiya M., Yamazaki A., Takashima E., Park J.W., Ueno K., Seki S., Serizawa N. (2013). Solvate Ionic Liquid Electrolyte for Li-S Batteries. J. Electrochem. Soc..

[10] Galinski M., Lewandowski A., Stepniak I. (2006). Ionic Liquids as Electrolytes. Electrochim. Acta.

[11] MacFarlane D.R., Forsyth S.A., Golding J., Deacon G.B. (2002). Ionic Liquids Based on Imidazolium, Ammonium and Pyrrolidinium Salts of the Dicyanamide Anion. Green Chem..

[12] Wang B., Qin L., Mu T., Xue Z., Gao G. (2017). Are Ionic Liquids Chemically Stable?. Chem. Rev..

[13] Zhou Z.B., Matsumoto H., Tatsumi K. (2005). Low-melting, Low-viscous, Hydrophobic Ionic Liquids: Aliphatic Quaternary Ammonium Salts with Perfluoroalkyltrifluoroborates. Chem. Eur. J..

[14] Agostini M., Sadd M., Xiong S.Z., Cavallo C., Heo J., Ahn J.H., Matic A. (2019). Designing a Safe Electrolyte Enabling Long-life Li/S Batteries. Chemsuschem.

[15] Suriyakumar S., Kathiresan M., Stephan A.M. (2019). Charge-discharge and Interfacial Properties of Ionic Liquid-added Hybrid Electrolytes for Lithium-Sulfur Batteries. ACS Omega.

[16] Yan Y., Yin Y.X., Guo Y.G., Wan L.J. (2014). Effect of Cations in Ionic Liquids on the Electrochemical Performance of Lithium-Sulfur Batteries. Sci. China Chem..

[17] Park J.W., Yamauchi K., Takashima E., Tachikawa N., Ueno K., Dokko K., Watanabe M. (2013). Solvent Effect of Room Temperature Ionic Liquids on Electrochemical Reactions in Lithium-Sulfur Batteries. J. Phys. Chem. C.

[18] Yang Y.B., Men F., Song Z.P., Zhou Y.H., Zhan H. (2017). N-methoxyethyl-N-methylpyrrolidinium Bis(trifluoromethanesulfonyl) Imide Ionic Liquid Based Hybrid Electrolyte for Lithium Sulfur Batteries. Electrochim. Acta.

[19] Lin Z., Liu Z.C., Fu W.J., Dudney N.J., Liang C.D. (2013). Phosphorous Pentasulfide as a Novel Additive for High-Performance Lithium-Sulfur Batteries. Adv. Funct. Mater..

[20] Zhang H., Eshetu G.G., Judez X., Li C.M., Rodriguez-Martinez L.M., Armand M. (2018). Electrolyte Additives for Lithium Metal Anodes and Rechargeable Lithium Metal Batteries: Progress and Perspectives. Angew. Chem. Int. Ed..

[21] Li W.Y., Yao H.B., Yan K., Zheng G.Y., Liang Z., Chiang Y.M., Cui Y. (2015). The Synergetic Effect of Lithium Polysulfide and Lithium Nitrate to Prevent Lithium Dendrite Growth. Nat. Commun..

[22] Zhang S.S. (2016). A New Finding on the Role of LiNO_3_ in Lithium-Sulfur Battery. J. Power Sources.

[23] Xiong S.Z., Kai X., Hong X.B., Diao Y. (2012). Effect of LiBOB as Additive on Electrochemical Properties of Lithium-Sulfur Batteries. Ionics.

[24] Kim S., Kwon Y.M., Cho K.Y., Yoon S. (2021). Metal Iodides (LiI, MgI_2_, AlI_3_, TiI_4_, and SnI_4_) Potentiality as Electrolyte Additives for Li-S Batteries. Electrochim. Acta.

[25] Rui L., Sun X.G., Zou J.Y., Qiang H. (2020). Lithium Fluoride as an Efficient Additive for Improved Electrochemical Performance of Li-S Batteries. Colloids Surf. A.

[26] Wu F., Zhu Q.Z., Chen R.J., Chen N., Chen Y., Ye Y.S., Qian J., Li L. (2015). Ionic Liquid-Based Electrolyte with Binary Lithium Salts for High Performance Lithium-Sulfur Batteries. J. Power Sources.

[27] Seong M.J., Yim T. (2021). Ionic Additives to Increase Electrochemical Utilization of Sulfur Cathode for Li-S Batteries. J. Electrochem. Sci. Technol..

[28] Sun J.P., Zhang K., Fu Y.Z., Guo W. (2023). Benzoselenol as an Organic Electrolyte Additive in Li-S Battery. Nano Res..

[29] Wang Y.J., Meng Y., Zhang Z.K., Guo Y., Xiao D. (2021). Trifunctional Electrolyte Additive Hexadecyltrioctylammonium Iodide for Lithium-Sulfur Batteries with Extended Cycle Life. ACS Appl. Mater. Interfaces.

[30] Vu D.L., Kim N., Myung Y., Yang M., Lee J.W. (2020). Aluminum Phosphate as a Bifunctional Additive for Improved Cycling Stability of Li-S Batteries. J. Power Sources.

[31] Lu H., Zhu Y., Yuan Y., He L., Zheng B., Zheng X.Z., Liu C.C., Du H.L. (2021). LiFSI as a Functional Additive of the Fluorinated Electrolyte for Rechargeable Li-S Batteries. J. Mater. Sci. Mater. Electron..

[32] Ji X., Lee K.T., Nazar L.F. (2009). A highly ordered nanostructured carbon-sulphur cathode for lithium-sulphur batteries. Nat. Mater..

[33] Wei J., Jiang C., Chen B., Li X., Zhang H. (2021). Hollow C/Co_9_S_8_ hybrid polyhedra-modified carbon nanofibers as sulfur hosts for promising Li-S batteries. Ceram. Int..

[34] Chen R., Shen J., Chen K., Tang M., Zeng T. (2021). Metallic phase MoS_2_ nanosheet decorated biomass carbon as sulfur hosts for advanced lithium-sulfur batteries. Appl. Surf. Sci..

[35] Howlett P.C., Izgorodina E.I., Forsyth M., MacFarlane D.R. (2006). Electrochemistry at Negative Potentials in Bis(trifluoromethanesulfonyl)amide Ionic Liquids. Z. Phys. Chem..

[36] Li Z., Borodin O., Smith G.D., Bedrov D. (2015). Effect of Organic Solvents on Li^+^ Ion Solvation and Transport in Ionic Liquid Electrolytes: A Molecular Dynamics Simulation Study. J. Phys. Chem. B.

[37] Oldiges K., Diddens D., Ebrahiminia M., Hooper J.B., Cekic-Laskovic I., Heuer A., Bedrov D., Winter M., Brunklaus G. (2018). Understanding Transport Mechanisms in Ionic Liquid/Carbonate Solvent Electrolyte Blends. Phys. Chem. Chem. Phys..

[38] Azimi N., Weng W., Takoudis C., Zhang Z.C. (2013). Improved Performance of Lithium-Sulfur Battery with Fluorinated Electrolyte. Electrochem. Commun..

[39] Busche M.R., Adelhelm P., Sommer H., Schneider H., Leitner K., Janek J. (2014). Systematical Electrochemical Study on the Parasitic Shuttle-Effect in Lithium-Sulfur-Cells at Different Temperatures and Different Rates. J. Power Sources.

[40] Barghamadi M., Best A.S., Bhatt A.I., Hollenkamp A.F., Mahon P.J., Musameh M., Ruther T. (2015). Effect of Anion on Behaviour of Li-S Battery Electrolyte Solutions Based on N-Methyl-N-Butyl-Pyrrolidinium Ionic Liquids. Electrochim. Acta.

[41] Barghamadi M., Best A.S., Bhatt A.I., Hollenkamp A.F., Mahon P.J., Musameh M., Ruther T. (2015). Effect of LiNO_3_ Additive and Pyrrolidinium Ionic Liquid on the Solid Electrolyte Interphase in the Lithium Sulfur Battery. J. Power Sources.

[42] Zhang Z.J., Zhang P., Liu Z.J., Du B.Y., Peng Z.Q. (2020). A Novel Zwitterionic Ionic Liquid-Based Electrolyte for More Efficient and Safer Lithium-Sulfur Batteries. ACS Appl. Mater. Interfaces.

