# Supplementary material for: An Ionic Liquid Electrolyte Additive for High-Performance Lithium–Sulfur Batteries
Source: Materials (Basel). 2023 Dec 4;16(23):7504. doi: 10.3390/ma16237504 (PMC10707666; doi:10.3390/ma16237504)
Supplement: Supplementary file 1 [file materials-16-07504-s001.zip › materials-2729206-supplementary.pdf]

materials

Support Information

An Ionic Liquid Electrolyte Additive for High-Performance Lithium Sulfur Battery

Zeliang Guan, Ling Bai, and Binyang Du *

MOE Key Laboratory of Macromolecular Synthesis and Functionalization, Department of Polymer Science & Engineering, Zhejiang University, Hangzhou 310027, China; 21929001@zju.edu.cn (Z.G.); 12129056@zju.edu.cn (L.B.)

* Correspondence: duby@zju.edu.cn

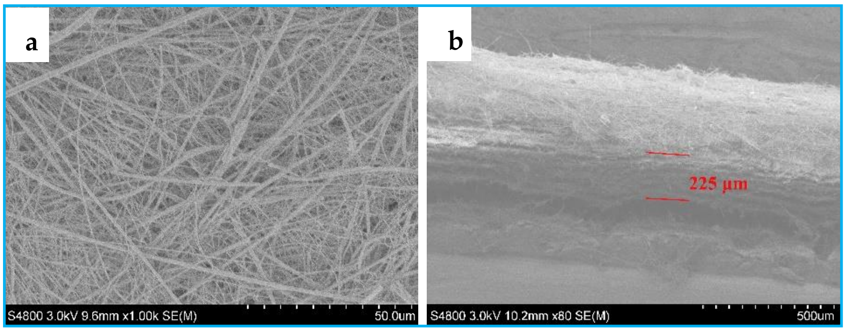

Figure S1. (**a**) SEM images of the top surface for glass fiber separator, (**b**) Cross-section of the glass fiber separator.

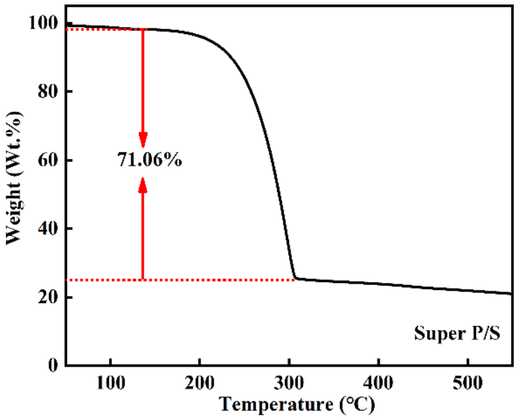

Figure S2. The TG curve of Super P/S.

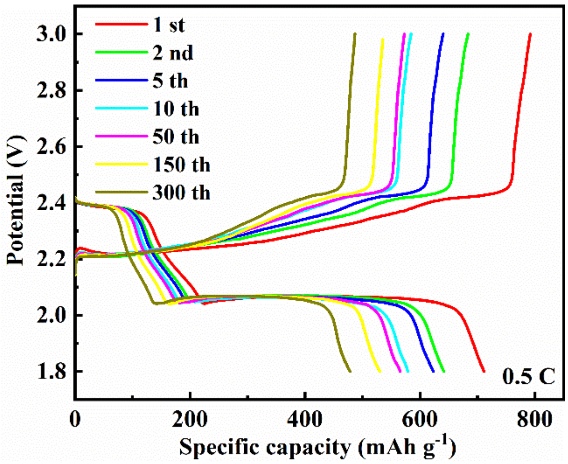

Figure S3. The charge-discharge curve of LSBs used electrolyte with 0% TDA+TFSI additive in different cycle, the current density is 0.5 C.
